# Transcriptomic insights into the dominance of two phototrophs throughout the water column of a tropical hypersaline-alkaline crater lake (Dziani Dzaha, Mayotte)

**DOI:** 10.3389/fmicb.2024.1368523

**Published:** 2024-04-29

**Authors:** Sébastien Duperron, Sébastien Halary, Jean-Pierre Bouly, Théotime Roussel, Myléne Hugoni, Maxime Bruto, Philippe M. Oger, Charlotte Duval, Anthony Woo, Didier Jézéquel, Magali Ader, Christophe Leboulanger, Hélène Agogué, Vincent Grossi, Marc Troussellier, Cécile Bernard

**Affiliations:** ^1^UMR 7245 MCAM, Muséum National d’Histoire Naturelle – CNRS, Paris, France; ^2^Université Claude Bernard Lyon 1, CNRS, INSA de Lyon, UMR 5240 Microbiologie Adaptation et Pathogénie, University of Lyon, Villeurbanne, France; ^3^Institut Universitaire de France, Paris, France; ^4^Anses, UMR Mycoplasmoses Animales, VetAgro Sup, Université de Lyon, Marcy-l’Étoile, France; ^5^Pôle Analyse de Données UAR 2700 2AD, Muséum National d’Histoire Naturelle, Paris, France; ^6^Institut de Physique du Globe de Paris, Université de Paris Cité, CNRS, Paris, France; ^7^UMR CARRTEL, INRAE-USMB, Thonon-les-Bains, France; ^8^MARBEC, Univ Montpellier, CNRS, Ifremer, IRD, Sète, France; ^9^Littoral Environnement et Sociétés, UMR 7266, CNRS La Rochelle Université, La Rochelle, France; ^10^LGL-TPE, UMR 5276, CNRS, ENSL, Université Claude Bernard Lyon 1, Villeurbanne, France

**Keywords:** *Limnospira platensis*, *Picocystis salinarum*, metatranscriptomics, photosynthesis, fermentation, transcriptomics

## Abstract

Saline-alkaline lakes often shelter high biomasses despite challenging conditions, owing to the occurrence of highly adapted phototrophs. Dziani Dzaha (Mayotte) is one such lake characterized by the stable co-dominance of the cyanobacterium *Limnospira platensis* and the picoeukaryote *Picocystis salinarum* throughout its water column. Despite light penetrating only into the uppermost meter, the prevailing co-dominance of these species persists even in light- and oxygen-deprived zones. Here, a depth profile of phototrophs metatranscriptomes, annotated using genomic data from isolated strains, is employed to identify expression patterns of genes related to carbon processing pathways including photosynthesis, transporters and fermentation. The findings indicate a prominence of gene expression associated with photosynthesis, with a peak of expression around 1 m below the surface, although the light intensity is very low and only red and dark red wavelengths can reach it, given the very high turbidity linked to the high biomass of *L. platensis*. Experiments on strains confirmed that both species do grow under these wavelengths, at rates comparable to those obtained under white light. A decrease in the expression of photosynthesis-related genes was observed in *L. platensis* with increasing depth, whereas *P. salinarum* maintained a very high pool of *psb*A transcripts down to the deepest point as a possible adaptation against photodamage, in the absence and/or very low levels of expression of genes involved in protection. In the aphotic/anoxic zone, expression of genes involved in fermentation pathways suggests active metabolism of reserve or available dissolved carbon compounds. Overall, *L. platensis* seems to be adapted to the uppermost water layer, where it is probably maintained thanks to gas vesicles, as evidenced by high expression of the *gvp*A gene. In contrast, *P. salinarum* occurs at similar densities throughout the water column, with a peak in abundance and gene expression levels which suggests a better adaptation to lower light intensities. These slight differences may contribute to limited inter-specific competition, favoring stable co-dominance of these two phototrophs.

## 1 Introduction

Saline-alkaline lakes are regarded as extreme environments due to the combination of high pH with high salinity levels. Those ecosystems harbor low species richness and diversity with up-to-exclusive dominance of microorganisms, and limited exchange with taxa from surrounding environments (Jones et al., 1994, 1998; Sorokin et al., 2014). In spite of a microbial diversity restricted to highly adapted microorganisms, some of these lakes are among the most productive ecosystems on Earth, in the range of kilograms per square meter and year, equivalent to the productivity of some forest ecosystems (e.g., Oduor and Schagerl, 2007). Intense biomass production in saline-alkaline lakes is related to high temperatures, high levels of light irradiation, and occurrence of well-adapted primary producers that benefit from high dissolved inorganic carbon concentrations, while suffering only limited competition and weak top-down control by predation (Burian et al., 2023). Indeed, semi-permanent to permanent blooms of phototrophs are often reported, in contrast to the more occasional blooms commonly observed in regular freshwater lakes (Krienitz and Schagerl, 2016).

Lake Dziani Dzaha is a volcanic crater lake located on the “Petite Terre” island of Mayotte (Leboulanger et al., 2017). It is a small (25 ha), shallow lake (∼3.5–4 m depth) over most of its surface, while a circular pit reaches 18 m depth on its Eastern part. Lake Dziani Dzaha has been proposed as one of the rare contemporary analogs of specific Precambrian environments owing to the paucity (possibly absence) of aquatic metazoans, and its physico-chemical characteristics (Cadeau et al., 2022). The biogeochemical functioning of the lake has remained overall stable over the 2010–2020 decade, with alternance of stratified and non-stratified periods, while some ongoing changes are documented since 2021. During the 2010–2020 period, the lake salinity exceeded 60 psu, its pH was above 9 and it presented seasonal sulfidic bottom waters and important methane production (Cadeau et al., 2020; Sarazin et al., 2020). Previous studies based on culture approaches or amplicon sequencing have described the composition and diversity patterns of lake Dziani Dzaha microbial communities (Hugoni et al., 2018). Consistent with findings in other east African lakes, the dominant phototroph and primary producer was a filamentous cyanobacterium belonging to genus *Limnospira* (formerly known as *Arthrospira*) (Oduor and Schagerl, 2007; Krienitz et al., 2012; Roussel et al., 2023). *L. platensis* (formerly *A. fusiformis*) represented 99% of the cyanobacteria according to high-throughput sequencing, and 97–99% in terms of biomass (Bernard et al., 2019). Interestingly, co-dominance of a second phototroph was observed in lake Dziani Dzaha in terms of cell numbers, namely with the picoeukaryote *Picocystis salinarum* (Chlorophyta), representing 99% of eukaryotic phytoplankton sequences based on rRNA genes-based metabarcoding approaches (Cellamare et al., 2018; Hugoni et al., 2018; Bernard et al., 2019). Due to its much smaller size though, its biomass contribution was negligible compared to that of *L. platensis*. However, the stable and exclusive co-dominance of these two phototrophs questions their respective functional strategies to cope with high pH and salinity conditions, and to avoid competitive exclusion (Bernard et al., 2019). Dominance of phototrophs is observed over the whole water column in other hypersaline lakes, even in the anoxic and low light environment below the euphotic zone (Phillips et al., 2021). Although cyanobacteria are oxygenic phototrophic organisms, they often thrive in anoxic environments. To do so, cyanobacteria generally utilize endogenous storage carbohydrates such as glycogen, and under dark anoxic conditions, energy generation potentially involves fermentation pathways (Stal and Moezelaar, 1997; Cellamare et al., 2018). Some species also have the ability to perform anoxygenic photosynthesis using elemental sulfide as an alternative electron donor in environments containing high sulfide concentrations or fermentative metabolism coupled with sulfur reduction in dark condition (Cohen et al., 1986; Oren and Shilo, 1979).

Despite their potential significance, functional shifts in phototroph metabolisms have been poorly explored in the water column of saline-alkaline lakes (Phillips et al., 2021). In this study, the main functions that may sustain *Limnospira platensis* and *Picocystis salinarum* co-dominance over the entire water column of lake Dziani Dzaha, including aphotic and anoxic zones, are explored. A metatranscriptomic approach addressing gene expression is used to identify the metabolism expressed by the two co-dominant phototrophs throughout the water column. We hypothesize that different abilities to acclimatize to local microhabitats may explain the vertical distribution of the two lineages. Thus, we explore the expression of the genes involved in photosynthetic processes across the water column and also test whether fermentation, using endogenous or exogenous organic substrates, could be an alternative metabolism enabling phototrophs to remain active in aphotic and anoxic zones (Phillips et al., 2021; Singh et al., 2023). The vertical distribution of gene expression levels is then investigated with a focus on transcripts involved in photoautotrophy, heterotrophic processes, and membrane-bound transporters. This study provides the first insights into the functioning of phototrophs in the water column of Lake Dziani Dzaha.

## 2 Materials and methods

### 2.1 Study site, sampling, and environmental parameters

Samples were collected from lake Dziani Dzaha (12°46′15.6″S; 45°17′19.2″E) in November 2017. Lake depth is 3.5–4 m in average, with a narrow pit located in the eastern part that reaches 18 m. This pit was sampled in order to cover the whole range of the water column. Water was collected on a depth profile at this deepest point of the lake (0, 1, 2.5, 5, 11, 14, and 16 m depth), using a horizontal 1.2 L Niskin bottle. From a depth, 220 mL of water was collected and immediately added with 220 mL of RNA later (ammonia sulfate 7.93 M, sodium citrate 0.025 M, EDTA 0.02 M, pH 5.2) to preserve RNA for metatranscriptomics. Samples were kept in the dark in a cool box and processed within 24 h following field sampling for microbiological analyses. Gradual filtration was performed by prefiltering 60 mL of water through 3 μm pore-size polycarbonate filters (Millipore) and then 30 mL of filtrates were passed through 0.2 μm pore-size polycarbonates filters (Millipore, pressure < 10 kPa). To collect enough biomass for to metatranscriptomics, the procedure was performed 7 times. All filters (3 and 0.2 μm) were conserved in liquid nitrogen and then stored at −80°C until RNA extraction.

Water column profiles for pH, dissolved O_2_, temperature and conductivity were acquired using either a MPP350 probe connected to a Multi 350i data logger (WTW GmbH) or a YSI 6600 probe. Salinity was calculated from conductivity and temperature measurements. The concentrations of soluble sulfide (ΣS(-II), hereafter referred to H_2_S/HS^–^), ammonium and ammonia (ΣN(-III); NH_4_^+^/NH_3_), and soluble-reactive phosphorus (SRP; PO_4_^3–^) were determined by colorimetry using Aqualytic SpectroDirect spectrophotometer and Merck reagents kits. The concentration of chlorophyll *a* (Chl *a*) was measured after extraction using 96% ethanol by ultra-sonication in an ice bath for 30′ seconds, and further extraction was allowed overnight (4 °C, dark). The extract was filtered, and full absorbance spectra (400–800 nm) recorded on clarified ethanol extracts. The concentration of Chl *a* was calculated according to a classic protocol (Ritchie, 2006).

### 2.2 *Limnospira* and *Picocystis* cell abundances and biomasses

Details regarding methodological procedures were described in Bernard et al. (2019). Briefly, for *Picocystis*, sub-samples (1.6 mL) from each depth were preserved using 80 μL of 0.2 μm-filtered 37% formaldehyde solution and stored in liquid nitrogen. In the lab, these were analyzed using a FACSAria Flow cytometer (Becton Dickinson, San Jose, CA, USA) equipped with the HeNe air-cooled laser (633 nm, 20 mW) as previously described (Bernard et al., 2019). Sub-samples of the lake water were collected at each sampling depth for microscopic examination, identification, and measurement of cyanobacterial cells. The samples were fixed (5% formaldehyde final concentration) and the taxa were identified as described previously (Cellamare et al., 2018). The cyanobacterial count data were obtained by the Utermöhl method using an Eclipse TS100 inverted microscope at 600× magnification (Nikon Instruments Inc., Melville, NY, USA) and biomass was expressed in μm^3^ ml^–1^ by using the cell biovolume of *Limnospira* and *Picocystis* (235.26 and 10.02 μm^3^, respectively) calculated as described previously (Catherine et al., 2012).

### 2.3 Strains isolation and growth rate experiment

Strains of *Limnospira platensis* PMC (Paris Museum Collection) 851.14, 894.15 and 917.15 were, respectively, isolated from a stromatolite biofilm and at 0.25 m depth of the water column of Lake Dziani Dzaha. Strains of *Picocystis salinarum* ALCP (Algotheque Laboratoire Cryptogamie Paris) 144.1, 145.1 and 146.1 were isolated at 0.25 m depth of the water column of Lake Dziani Dzaha (Cellamare et al., 2018; Bernard et al., 2019). Isolated strains and cultures were all monoclonal and non-axenic.

The cultures of *L. platensis* and *P. salinarum* were carried out in 30 mL of Z8 medium (Rippka, 1998) with nitrate (NO_3_**^–^**) as the nitrogen source, in 50 mL flasks of 2.5 cm width, inoculated from a solution optical density at 750 nm (OD_750_) of 0.1 (spectrophotometer Cary 60 Agilent Technologies). Triplicates of each culture conditions were run, and were manually shaken twice daily to keep the cells in suspension (15 μ mol photons s**^–^**^1^m**^–^**^2^; 16:8 light:dark).

Cultures were done under white, blue, green and red lights using light-emitting diodes (LEDs). The wavelengths of the emission peaks were measured using a spectroradiometer (JAZ Ocean Optics) and were of λ_Blue_ = 464 ± 23 nm, λ_Green_ = 521 ± 32 nm and λ_Red_ = 633 ± 17 nm. The white light spectrum was from 400 to 700 nm, partly similar to the spectrum of sunlight. The data were processed with the Spectrasuite software.

The growth curves of the six strains were produced from the OD_750_ measurements, transformed into cell number using the OD_750_—cell density calibration curves (Supplementary Figure 1). The logarithmic transformation made it possible to linearize the exponential growth phase, then to calculate a maximum growth rate (μ_max_) [d^–1^], equivalent to the slope of the regression line. Statistical analyses of growth rates were done using GraphPad software. Analyzes of covariance (ANCOVA) with Tukey’s *post-hoc* tests, were carried out to compare the slopes of the linear regressions.

### 2.4 RNA extraction, metatranscriptomics library preparation and sequencing

For both 3 and 0.2 μm filters, RNA was extracted separately from three filters (i.e., three replicates per sample) using the Quick-RNA™ MiniPrep kit (Zymo Research) optimized for hypersaline samples. Each filter was cut in very thin sections using sterile material before introduction in 2 mL ZR Bashing Bead Lysis Tubes in which 500 μL of phenol:chloroform:isoamyl alcohol (25: 24: 1) and 600 μL of Lysis buffer from the kit were added. Samples were then homogenized using a FastPrep-24™ 5G (MP Biomedicals™) at 6 m.sec^–1^ (30 s), placed on ice (5 min), and homogenized again (6 m.sec^–1^, 30 s). Centrifugation was performed (5 min, 13,000 g, 4°C). Then the aqueous phase was transferred to step 2 of the Quick-RNA™ MiniPrep kit, following manufacturer’s instructions. Elution was performed using two 100 μL volumes of DNase/RNase-Free water that were later combined. DNAse treatment was performed using TURBO DNA-*free*™ kit (Invitrogen™) and a generalist 16S rRNA genes PCR was realized using 515F/909R primers (Parada et al., 2016; Luis et al., 2019) to test for the absence of residual DNA contamination. RNA extracts from triplicates were then pooled and quantified using the Qubit RNA BR Assay Kit (Invitrogen, Carlsbad, USA) following the manufacturer’s instructions and quality was evaluated using a Bioanalyzer Pico chip (Agilent). The RNA extracts were stored at −80°C until library preparation.

Library was produced after RiboZero Gold + Bacterial kit (for rRNA depletion), using the TruSeq stranded mRNA kit and sequencing was achieved on HiSeq 4000 2*150 bp (on average 40M read-pairs expected, Fasteris company, Plan-les-Ouates, Switzerland).

### 2.5 *Limnospira* and *Picocystis* genomes reconstruction from cultures

DNA was extracted from cultures of isolated strains of *L. platensis* PMC 851.14 and *P. salinarum* ALCP 144.1 using a ZymoBIOMICS DNA mini kit (Zymo Research, CA, USA) from strains grown in flasks. Mechanical lysis was carried out using an ultrasonic cell probe Vibra Sonic (Granuloshop) for 30 s at a range of 100% to 32.5 W. Total DNA was sequenced using both Illumina HiSeq 2 × 250 bp and SMRT cell PacBio RS2 platforms (Genoscreen, France). Scaffolds were assembled from MiSeq and Pacbio reads using SPAdes-based Unicycler hybrid-assembler, with default parameters (Bankevich et al., 2012; Wick et al., 2017). To refine genomic assemblies by discarding genomic sequences from heterotrophic bacteria growing in the non-axenic cultures of both phototrophs, a binning step was conducted. To this aim, scaffolds coverages were assessed by mapping back the Illumina sequencing reads on assembled sequences using Bowtie2 (Langmead and Salzberg, 2012) and calculation using Samtools and custom bash scripts (Li et al., 2009). In the case of *L. platensis* associated metagenome, binning was then performed using MyCC coupled to Prodigal to predict open reading frame (ORF)s (Hyatt et al., 2010; Lin and Liao, 2016), and resulting bins were taxonomically annotated using CAT (von Meijenfeldt et al., 2019). Genes carried by genomic sequences from *L. platensis* bin were finally functionally annotated using Eggnog-mapper v2 (Huerta-Cepas et al., 2019).

In the case of *P. salinarum*, chloroplastic and mitochondrial genomes were directly retrieved in the assembly as they were the only two complete circular sequences and their nature was confirmed by aligning them against the *nt* database using megablast (Altschul et al., 1990). The nuclear genomic region were then extracted after binning carried out by Anvi’o (Eren et al., 2015). Prediction of *P. salinarum* transcripts was performed on nuclear and mitochondrial genomes using GeneMarkES and functionally annotated by Eggnog mapper (Huerta-Cepas et al., 2019) while chloroplastic CDS was predicted and annotated using GeSeq (Tillich et al., 2017). Both genomes completeness and contamination were assessed using CheckM (Parks et al., 2015).

### 2.6 Metatranscriptomic analysis

Paired-end HiSeq reads were quality checked and trimmed using *fastx toolkit* (Gordon, 2010). For each metatranscriptome, filtered reads corresponding to *L. platensis* or *P. salinarum* were retrieved by mapping them against respective MAGs using *bbsplit* (Bushnell, 2014). Paired-end transcript fragments were finally aligned against the species corresponding transcripts database using Salmon using default parameters (Patro et al., 2017). Expression levels were normalized as transcripts per kilobase million (TPM) for both phototrophs at each given depth.

## 3 Results

### 3.1 Chemistry, phytoplankton cell abundances and biomass in the water column

**FIGURE 1:**
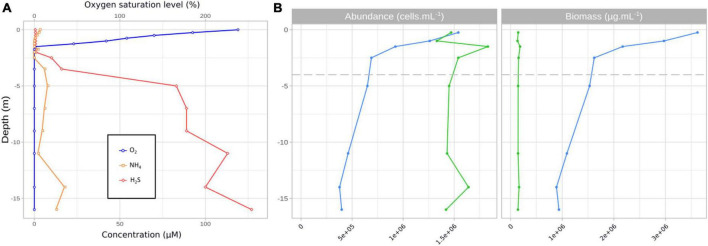
Oxygen, H_2_S and NH_4_ profiles in the water column at the sampling date DZ11-17. Numbers above the *x*-axis refer to oxygen saturation level (%), numbers below the *x*-axis refer to concentrations (μM) **(A)**. Cell abundances (cells mL^–1^, left) and estimated biomass (μg C mL^–1^, right) of *Limnospira platensis* (blue) and *Picocystis salinarum* (green) in the water column **(B)**.

At the time of sampling, the lake water column was characterized by a strong gradient of oxygen (from 238% saturation at the surface to 0% below 1.5 m, Figure 1A) corresponding to the oxic zone. The photic zone corresponded to the first 0.5 m depth. A strong gradient of reduced sulfur (H_2_S/HS^–^) was also observed with values < 1 μM from the surface down to 2.5 m and from 10 to 126 μM from 2.5 to 16 m (Figure 1A and Supplementary Table 1). Salinity was high and homogenous throughout the water column (63.9 ± 0.47 psu). Ammonium concentrations were high (3.42 to 0.55 μM NH_4_^+^) from the surface to 0.75 m, then dropped below the detection limit from 0.75 to 2.5 m (except for 1.75 m where the concentration was 2.2 μM) and then increased up to 17.9 μM at the lake bottom (Figure 1A and Supplementary Table 1). Dissolved organic carbon values were stable throughout the water column (6.77 to 7.64 mM).

Abundances estimates of *L. platensis* and *P. salinarum* displayed contrasting trends (Figure 1B and Supplementary Table 1). *P. salinarum* cell numbers were stable throughout the column (1.51 ± 0.16 10^6–^cells mL^–1^), varying by a factor of 1.1, while counts of *L. platensis* varied by a factor of 4.1 between the maximum and minimum values (1.54 10^6^ versus 3.77 10^5^ cells mL^–1^) measured at 0.25 and 14 meters depth, respectively. In terms of estimated biomass, *L. platensis* was always highly dominant, with a biomass ratio ranging from 24.6 (surface sample) to 4.6 (14 m depth) versus *P. salinarum* (Figure 1B).

### 3.2 Growth rate depending on light quality in culture experiments

**FIGURE 2:**
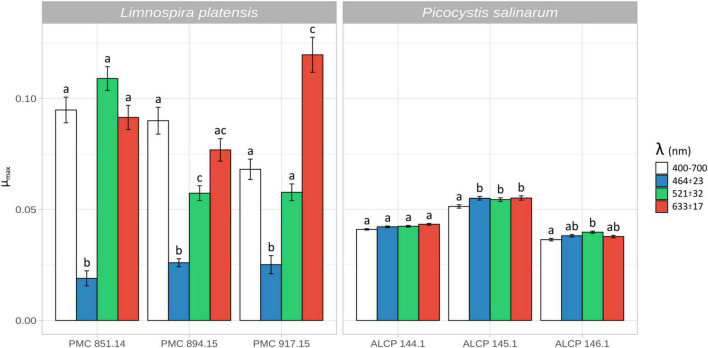
Maximum growth rates [d^–1^] for three strains of *Limnospira platensis* PMC 851.14, PMC 894.15, PMC 917.15 **(left)** and *Picocystis salinarum* ALCP 144.1, ALCP 145.1, ALCP 146.1 **(right)**, in monoculture, under white, blue, green and red light. Error bars are based on three replicate cultures for each strain and condition. Letters correspond to significantly different treatment groups (*p* < 0.05).

Experiments on isolated strains under laboratory light intensity (corresponding to 0.5% of intensities measured in Dziani Dzaha lake surface) showed that all *Limnospira platensis* strains displayed significantly (*p* < 0.001) lower growth levels when exposed to blue light (464 ± 23 nm) compared to white light (400–700 nm) (μ_max_ [d^–1^] = 0.023 ± 0.004 and 0.084 ± 0.014, respectively; Figure 2). While displaying varying responses to green light (521 ± 32 nm; μ_max_ [d^–1^] = 0.075 ± 0.030), all strains showed high growth rate under red light (633 ± 17 nm; μ_max_ [d^–1^] = 0.096 ± 0.022). Comparatively, growth rates ranged from lower (PMC 851.14) to higher (PMC 917.15) than those observed under white light (with *p*-values < 0.05), showing distinctive strain-specific differences in photoacclimation capacities (Supplementary Table 2).

Regardless of light quality, the growth rates of the *Picocystis salinarum* strains were overall comparable among conditions. Low yet significant differences were detected (*p*-values < 0.05), with lower growth rate in white versus other lights for strain ALCP 145.1, and in white versus green light for strain ALCP 146.1.

### 3.3 Reference genomes of the two strains *Limnospira platensis* (PMC 851.14) and *Picocystis salinarum* (ALCP 144.1)

The genome length of *L. platensis* PMC 851 is 6.07Mb, with 44.8% GC content. A partial nuclear genome was obtained from *P. salinarum*, displaying a total size of 16.84 Mb, yet with full chloroplast and mitochondrial genomes of 81.12 kb (Supplementary Figure 2) and 41.91 kb, respectively. In this study, these sequences were used for mapping metatranscriptome sequences.

### 3.4 *In situ* transcript abundances in the size fractions, and variation of most abundant transcripts with depth

Transcripts from *L. platensis* were highly dominant in the metatranscriptomic dataset obtained from the > 3 μm fraction, representing between 66.7 and 80.3% of total transcripts throughout the water column, while representing from 0.7 to 2.4% of total transcripts in the 3–0.2 μm fraction (Table 1). On the other hand, transcripts from the picoeukaryote *P. salinarum* were abundant in the 3–0.2 μm fraction, representing between 5.9 and 12.9% of total transcripts throughout the water column, and less abundant in the larger fraction (> 3 μm, up to 1.0%, Table 1). In both cases, no clear depth-related variation trend in transcript absolute of relative abundance was observed in the water column.

**TABLE 1:** Number of total reads, and number and fraction of reads (versus total reads) assigned to *Picocystis salinarum* and *Limnospira platensis* in the two size fractions (> 3 μm and 3–0.22 μm).

Depth (m)	Pore size (μ m)	Total reads	*Picocystis salinarum*		*Limnospira platensis*	
			Reads	% vs. total	Reads	% vs. total
0.25	3	69,227,568	192,554	0.3	55,571,197	80.3
1	3	86,744,204	223,468	0.3	68,488,093	79.0
2.5	3	65,972,220	650,989	1.0	44,023,208	66.7
5	3	61,851,945	455,417	0.7	45,742,074	74.0
11	3	66,195,995	441,656	0.7	47,805,510	72.2
14	3	75,836,235	277,987	0.4	55,402,335	73.1
16	3	73,623,140	366,566	0.5	54,806,389	74.4
0.25	0.2	41,314,879	3,571,092	8.6	436,748	1.1
1	0.2	37,438,714	4,216,066	11.3	414,152	1.1
2.5	0.2	49,165,349	6,319,214	12.9	434,503	0.9
5	0.2	39,691,029	3,040,611	7.7	708,356	1.8
11	0.2	48,277,891	2,855,226	5.9	782,848	1.6
14	0.2	43,144,610	4,110,663	9.5	301,934	0.7
16	0.2	43,836,078	4,500,373	10.3	1,046,337	2.4

**FIGURE 3:**
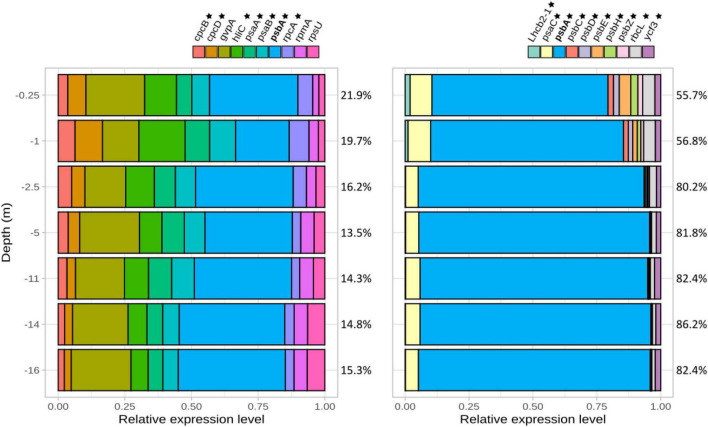
Most expressed genes assigned to *Limnospira platensis* **(left)** and *Picocystis salinarum* **(right)** normalized *versus* the total of these most expressed gene transcripts assigned to the respective species (i.e., 100% corresponding to the total expression level of these most expressed genes). Percentage of reads corresponding to these most expressed genes compared to the species total TPM are indicated for each depth on the right (e.g., the 10 most expressed genes correspond to 21.9% of *L. platensis* reads at 0.25 m depth). Genes involved in photosynthesis are labeled with a star. *psa*A, photosystem I P700 chlorophyll a apoprotein A1; *psa*B, photosystem I P700 chlorophyll a apoprotein A2; *psb*A, photosystem II protein D1; *psb*B, photosystem II CP47 reaction center protein; *psb*C, photosystem II CP43 reaction center protein; *psb*D, photosystem II D2 protein; *rbc*L, ribulose bisphosphate carboxylase large chain; *rbc*S, ribulose bisphosphate carboxylase small subunit, *Lhc*b2-1, light-harvesting chlorophyll binding.

Among transcripts attributed to *Limnospira platensis*, 10 genes were ranked as highly expressed over the water column (i.e., corresponding to the top 10 most expressed known genes in average over the whole water column). Seven of them were involved in photosynthesis, namely *cpc*B (phycocyanin beta chain), *cpc*D (phycocyanin associated linker protein), *rpc*A (R-phycocyanin II alpha chain), *psa*A (photosystem I P700 chlorophyll a apoprotein A1), *psa*B (photosystem I P700 chlorophyll a apoprotein A2), *hli*C (high-light inducible protein C associated to photosystem II) *ps*bA (photosystem II protein D1) (Figure 3, left); *psb*A was the most abundant transcript, with a minimum value at 2.5 m below which it tended to increase down to 16 m depth. The three highly expressed genes not directly related to photosynthesis included *gvp*A, coding for the protein A of cyanobacterial gas vesicles, essential for buoyancy and position of the cell in the water column (Supplementary Figure 3), and *rpm*A and *rps*U, both coding for ribosomal proteins. Photosynthesis-involved transcripts only decreased slightly in proportion with increasing water depth, and still represented the majority of transcripts of dominant genes at the deepest sampled point (16 m depth). For *Picocystis salinarum*, the dominance of photosynthetic gene transcripts was even higher, with all 10 most highly expressed genes involved in photosynthesis, and an overwhelming dominance of *psb*A (69.02 to 91.26% of the 10 most highly expressed transcripts, Figure 3, right).

### 3.5 Variation of photosynthesis genes expression with depth

**FIGURE 4:**
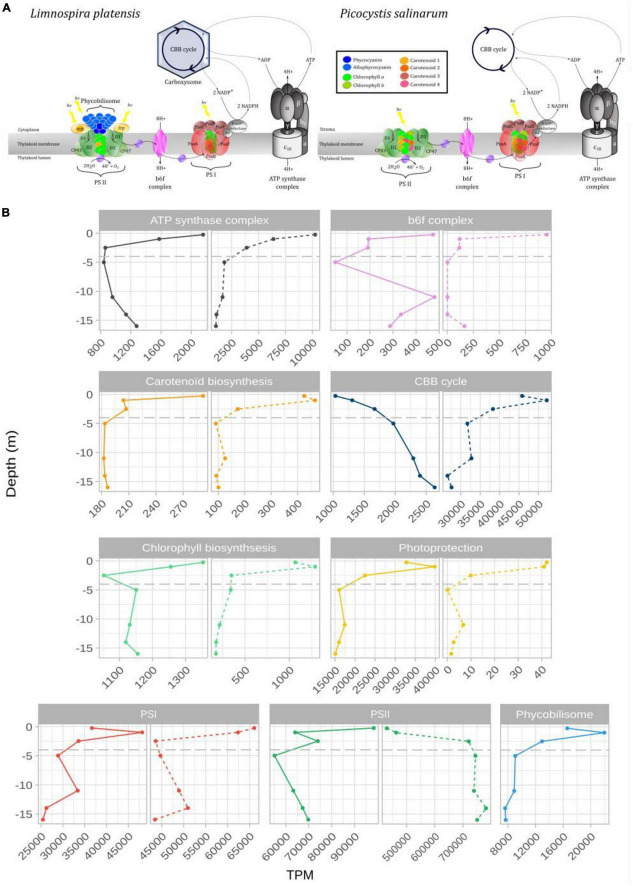
Schematic representation of light harvesting antenna and ATP synthase complex of *Limnospira platensis* and *Picocystis salinarum* **(A)**. **(B)** Expression levels (TPM: transcripts per kilobase million) of genes grouped by photosystem or complex for *L. platensis* (solid lines) and *P. salinarum* (dashed lines) versus depth. The detailed list of transcripts belonging to each group can be found in Supplementary Table 3.

Among the photosynthesis-related genes, assigned according to their role in the photosynthetic apparatus, gene expression (in TPM: transcripts per kilobase million from said phototroph) was dominated by genes involved in both photosystem I and II in *L. platensis* and in *P. salinarum* (Figures 4A, B and Supplementary Table 3). In the former species, a slight increase was observed in the expression of genes involved in photosystem I and phycobiliprotein biosynthesis between the surface and the first meter before a decrease until 2.5 meters depth, below which the levels were stable, representing 2.5–5% and around 0.8% of TPM (respectively) down to 16 meters depth (Figure 4B and Supplementary Table 3). Photosystem II-related genes expression level represented around 10% of TPM at 0.25 m of depth, then sharply decreased to 6.4% at 1 m, while values ranged between 5.5 and 7.4% in the deeper layers of the water column.

In *P. salinarum*, highest levels throughout the water column were recorded for photosystem II genes. Values increased from the surface down to the below the oxycline (2.5 m depth), below which they remained relatively stable, consistently representing over 70% of TPM. This trend is due to the high dominance of *psb*A which increased from surface to 2.5 m depth, while all other PSII genes decreased sharply (Supplementary Table 3). PS I genes expression levels decreased from surface to the oxycline, then remained stable representing around 5% of TPM down to the deepest points (Figure 4B). Expression of genes involved in carotenoids synthesis was very low throughout the water column. Expression levels of genes involved in photoprotection decreased sharply from surface to 2.5 m depth in both species, yet overall levels were 1000-fold lower in *P. salinarum* compared to *L. platensis* (Figure 4B).

### 3.6 Expression of fermentative pathways

**FIGURE 5:**
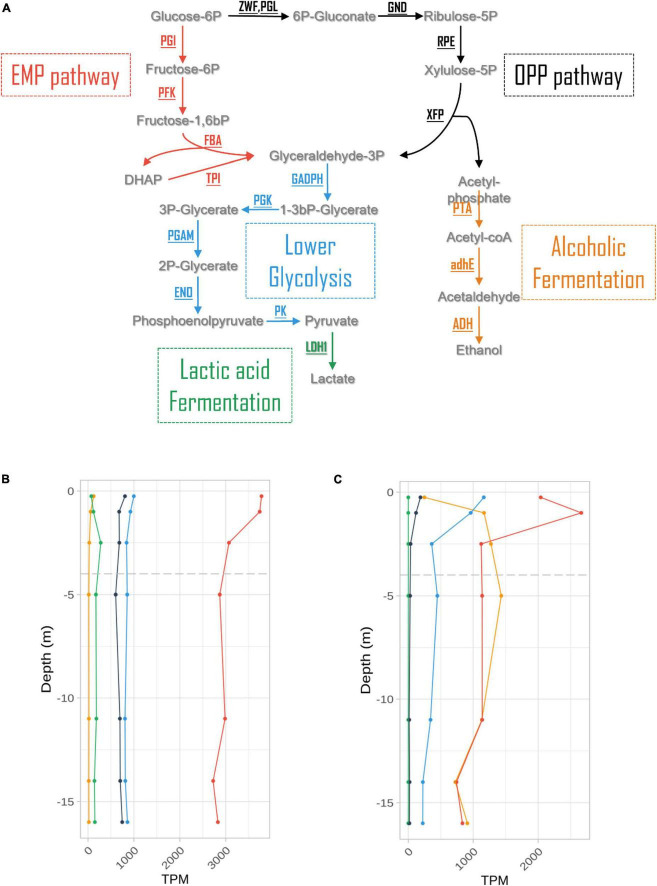
Expression levels (in TPM) of genes involved in the main fermentation pathways **(A)** in *Limnospira platensis* **(B)** and *Picocystis salinarum* **(C)** vs. depth.

Expression levels for genes involved in fermentative processes were extracted from the dataset at all depths (Figure 5 and Supplementary Table 3). These levels were one order of magnitude lower than those measured for photosynthesis in *L. platensis*, in which genes involved in fermentation processes represented a maximum of 0.37% of all transcripts. The glycolysis/gluconeogenesis pathway expression levels decreased over the first five meters of the water column, with a majority of transcripts associated with the Embden-Meyerhof-Parnas (EMP) pathway. Only the expression of lactic acid fermentation genes increased slightly with depth, reaching a maximum at 2.5 m (oxycline) before declining (Figure 5B).

Similar results were observed in *P. salinarum* for lower glycolysis and pentose phosphate (OPP) pathways. But other pathways showed marked differences with *L. platensis*. Firstly, although lactic acid fermentation genes have been characterized in the *P. salinarum* genome, no transcripts were found. Secondly, gene expression levels for the EMP pathway and alcoholic (ethanol) fermentation pathways increased with depth, reaching maxima at 1 and 5 m, respectively, 0.27 and 0.14% of transcripts, displaying similar values from the oxycline to 16 m depth (Figure 5C and Supplementary Table 3).

### 3.7 Expression of carbon compounds transporters in *L. platensis*

**FIGURE 6:**
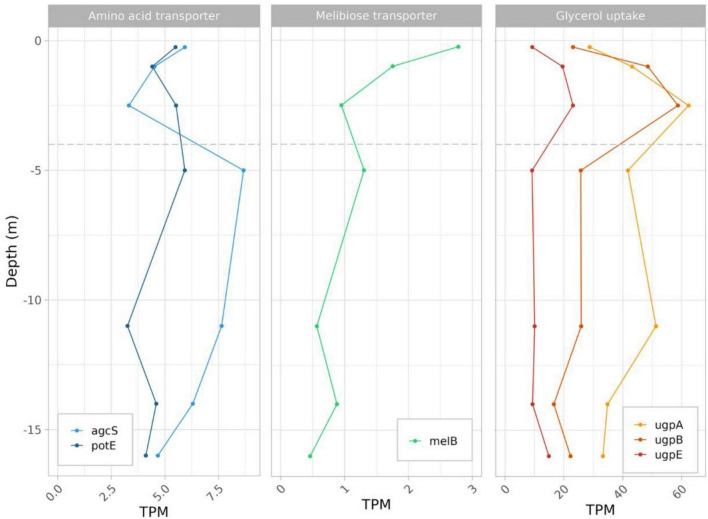
Expression level profiles of genes involved in amino acid, glucose/melibiose and glycerol uptake in *L. platensis*, vs. depth.

In *L. platensis*, transcripts from several transporters relevant to heterotrophic metabolism were identified, but representing a very low percentage of total transcripts. They included amino acid transporters Alanine or Glycine:Cation Symporter (*agc*S) for which expression level decreased sharply from subsurface to the oxycline below which it increased sharply at 5 m and then slowly decreased to the 16 m depth (Bualuang et al., 2015; Ma et al., 2019), and *pot*E (an amino-acid transporter) which was relatively stable throughout the water column (Figure 6 and Supplementary Table 3). Similarly, the melibiose transporter-encoding gene *mel*B transcript, documented to be a high-affinity glucose transporter in *Prochlorococcus* (Muñoz-Marín et al., 2017), decreased from subsurface to the oxycline above which it remained relatively stable (Figure 6). Glycerol transporters (*ugp*A, *B* and *E*) were also identified, with maximum expression levels located at the oxic:anoxic interface at 2.5 m depth (Figure 6).

Identifying transcripts relevant to carbon compound acquisition was not possible in *P. salinarum* because annotation doesn’t allow to establish whether a transporter is truly associated to the cell membrane, and thus relevant to uptake from the exterior instead of among internal eukaryotic cell compartments.

## 4 Discussion

Over the 2010–2020 period, the water column of Lake Dziani Dzaha consisted of a thin oxic layer, from the surface to ∼1.75 m depth, below which water was anoxic. The 1% photosynthetically available radiation (PAR) was located at ∼0.75 m below the surface (while average lake depth is ∼3.5–4 m) due to high turbidity and phototrophs biomass (Leboulanger et al., 2017). This extreme ecosystem was devoid of aquatic metazoans. High biomass of phytoplankton was observed consistently throughout the water column (e.g., mean_2014–2015_ = 652 ± 179 μg Chl*a* L^–1^), and the estimated productivity reached 3.0 10^3^ gC m^–2^ yr^–1^ [75 10^6^ gC yr^–1^ over an area of 25 ha, (Leboulanger et al., 2017)]. Upon sampling in 2017, the lake was very turbid with a permanent green color due to its high photosynthetic biomass. The water column revealed stable pH, temperature and high salinity throughout. However, the surface down to 1.75 meter displayed sharp decreasing gradient in oxygen, while reduced compounds (ammonium and sulfide) increased below the oxycline, confirming the occurrence of two very distinct habitats. A similar habitat partition was described based on previous sampling campaigns, suggesting that this stratification was observable on a regular, seasonal basis (Leboulanger et al., 2017; Hugoni et al., 2018; Sarazin et al., 2020; Escalas et al., 2021).

### 4.1 Abundances of the two dominant phototrophs display contrasted trends in the water column

The co-dominance of two phototrophic organisms in Lake Dziani Dzaha offers an opportunity for studying how functionally similar species thrive together despite potential competition. In 2017, *Limnospira platensis* abundances decreased with depth, contrasting with the stable presence of *Picocystis salinarum* across the water column, despite varying environmental conditions. Owing to their much larger size and volume, the former, however, always represented much higher biomass than the eukaryote. Previous work spanning April 2014 and November 2015, revealed comparable co-dominance in abundances (in terms of cell numbers) over the whole water column indicating a stable pattern (Bernard et al., 2019). Given the high water turbidity essentially due to *L. platensis* high biomass, only longer wavelengths can pass through the first centimeters below the surface (i.e., red and dark red light) (Hargreaves, 2003; Stomp et al., 2007). *P. salinarum* and *L. platensis* growth experiments suggest that both species can actively grow at low light intensities and under red light, which probably allow them to thrive in conditions documented across the first meter depth in lake Dziani. These results are in agreement with Stamps et al. (2018) who suggested that *Picocystis* was capable of photosynthesis under very low light conditions in the Mono Lake (California), including growth despite less than 0.1% of incident summertime solar irradiance as deep as 25 m below lake surface. Another study found temperatures between 30 and 40°C and salinities around 60 psu, comparable to those observed in lake Dziani Dzaha, to maximize the growth of a *P. salinarum* strain from Nakuru, Kenya (Pálmai et al., 2020).

### 4.2 Differences in photosynthesis-related genes expression levels throughout the water column

Gene expression was dominated by photosynthesis-related genes throughout the water column. In the first meter below the surface, expression levels are either maximal just below the surface, then sharply decrease (e.g., PSII, carotenoids and chlorophyll biosynthesis for *L. platensis*, or PSI for *P. salinarum*) or increase between the surface and the first meter (e.g., photoprotection, PSI, phycobiliprotein biosynthesis for *L. platensis*, or carotenoids and chlorophyll biosynthesis for *P. salinarum*). This maximum of various transcripts 1 m below the surface suggests sustained, possibly optimal photosynthetic activity there, where light intensity conditions in particular are congruent with aforementioned experiments performed on strains. Indeed, in uppermost surface layers (0.25 m), factors like high UV exposure, intense light, and excessive oxygen can hinder effective photosynthesis. Photoinhibition seems important for both species in this 0.25 m uppermost layer, as evidenced by the lower levels of CBB (Calvin Benson Bassham) cycle transcripts there. Below 1 meter depth, there is a general decrease in the expression levels of most photosynthesis genes. However, exceptions are observed in the case of *L. platensis* where the CBB genes maintain their expression, albeit with a limited increase of 1.5-fold. Conversely, for *P. salinarum*, while the *psb*A gene (protein D1, PSII) increases (explaining the overall increase observed for PSII), what stands out is its consistently high expression levels observed across the entire water column.

### 4.3 *Limnospira platensis* is primarily adapted to the upper water column

High expression levels measured in *Limnospira* for the *gvp*A gene, encoding a major gas vesicle structural protein (Supplementary Figure 3), suggest active control of cell buoyancy that possibly limits sinking and maintains higher abundances in upper waters (Damerval et al., 1991), possibly explaining *L. platensis* densities decrease from surface to deeper waters A consequence is that *L. platensis* is exposed to highest light levels. Cyanobacterial cells can trigger several fast (in the range of minutes) photoprotective mechanisms. The three most important strategies are photoinhibition, phycobilisome decoupling, and non-photochemical quenching (NPQ) (Kok, 1956; Adir et al., 2003; Kirilovsky, 2007). At the molecular level, PSII complexes are more sensitive to high light than PSI and the decrease in PSII activity is caused by degradation of the D1 from the reaction core center. In Dziani lake, among highly expressed genes, *psb*A encoding the D1 protein, is the most abundant transcript. As this protein is the primary target of photodamage, and is reportedly the most rapidly turning-over component of PSII exposed to light, this may explain its higher expression levels in the upper water column, as well as its decrease with depth (Wu et al., 2011).

### 4.4 *Picocystis salinarum* compensates the lack of photoprotection mechanisms by PSII repair

*Picocystis salinarum* is reportedly adapted to low photon flux, and tolerates absence of oxygen as well as high sulfide concentrations, allowing deeper survival in the lake (Roesler et al., 2002; Phillips et al., 2021). This is intriguing given the very high light levels observed in lake Dziani surface waters. Investigation of capacities for classical mechanisms involved in photoprotection indicated that the *Psb*S gene and LHCSr genes were not present in the *P. salinarum* genome, and that transcripts levels for violaxanthin de-epoxidase were very low despite the gene being present (Tibiletti et al., 2016; Perin et al., 2023). A first potential protective mechanism would be shading by neighbors, including the highly abundant *L. platensis*, which contributes to the absorption of a substantial amount of the light energy. Also, gene *psb*A from the chloroplast genome, encoding the D1 protein (Sirpiö et al., 2013), is by far the most expressed transcript throughout the water column. As for *L. platensis*, D1 is the most rapidly turning-over component of PSII, which may explain its very high expression levels compared to other components of the photosynthetic apparatus. One major difference with the prokaryote *L. platensis* is that mRNAs have much longer half-lives in eukaryotes, and regulation often occurs at the level of translation rather than transcription. Although *psb*A regulation is not yet documented in genus *Picocystis*, this gene is transcribed constitutively in higher plants and green algae including *Chlamydomonas*, leading to a stable transcripts pool, while translation is light-induced (Mayfield et al., 1994; Mulo et al., 2012). Massive production of *psb*A transcripts could thus be a way for *P. salinarum* to maintain this large pool of transcripts that can be translated on-demand when microalgae are exposed to higher light intensity and/or temperatures and D1 turn-over is maximal. This mechanism could compensate for the limited photoprotection mechanisms encoded by its genome in face of stressful environmental conditions. If confirmed, this strategy would represent a potential adaptation to hot hypersaline alkaline habitats that certainly warrants further study and experimental confirmation on laboratory experiments.

*P. salinarum* from lake Dziani Dzaha has a higher chlorophyll *a* content per volume compared to *L. platensis* [51.9 ± 12.8 versus 6.0 ± 4.9 fg.μm^–3^ (Bernard et al., 2019)]. *Picocystis* strain ML from the Mono Lake was also shown to harbor 10 times more chlorophyll *a* compared to other green algae (Roesler et al., 2002), and displayed high levels of transcription for photosynthesis-related genes down to 25 m depth suggesting a potential active photosynthesis under extremely low light conditions (Stamps et al., 2018). In Stamps et al.’s study, transcription levels were normalized versus the whole transcripts dataset, meanwhile values reported in this study are normalized versus *Picocystis* transcripts alone. When normalizing versus all transcripts, estimates for *psb*A abundances are still ∼40 times higher than those reported in this previous study. Such *psb*A expression levels are thus unexpectedly high, above 40% of *Picocystis*-affiliated transcripts from 2.5 m depth down. However, some peculiarities of *P. salinarum* in lake Dziani Dzaha might explain these levels. First, *Picocystis* photosystem-related proteins were shown to be strongly upregulated, with increased growth rates, at high pH, temperature and salinity, all three being present in lake Dziani Dzaha (Singh et al., 2023). Second, the stability of the D1 protein could be negatively affected by the very high light intensity and very high oxygen content in upper waters and the presence of H_2_S in the lower water, possibly further accelerating turn-over rates.

### 4.5 Fermentation for coping with the anoxic, aphotic zone

Certain Cyanobacteria such as *Pseudanabaena* strain FS39 can shift from oxygenic to sulfide-driven anoxygenic photosynthesis, but we found neither evidence for the presence in the genome nor for expression of the gene encoding sulfide quinone oxidoreductase (SQR), responsible for the oxidation of sulfide to sulfur and electron transfer to photosystem I (PSI), in *L. platensis*. Because this gene is present in all characterized cyanobacteria that perform anoxygenic photosynthesis, it suggests that *L. platensis* is not using this metabolism (Arieli et al., 1994; Hamilton et al., 2018). However, both species express genes for several fermentation pathways throughout the water column. Chemoorganotrophic growth is documented in some strains of *L. platensis* (Stal and Moezelaar, 1997). The sustenance of this heterotrophic metabolism finds potential support from the high concentrations of dissolved organic carbon that were recently reported in the euphotic layer of the lake Dziani Dzaha [i.e., 3–9 mMol L^–1^, (Sarazin et al., 2020)] as well as in this study (6.77 to 7.64 mM). Also, glycogen is reportedly bioaccumulated in *Limnospira* species (Cuellar-Bermudez et al., 2022). Additionally, the possible presence of a phycosphere surrounding *L. platensis* filaments (Bruto et al., 2023) may also contribute and would be congruent with the expression of several amino acid, sugar and glycerol transporters found in the present study throughout the water column. Interestingly, highest levels of expression for glycerol uptake genes are observed right below the oxic to anoxic transition zone (2.5 m, the first sampling depth located below the oxycline). Among fermentation pathways, the EMP pathway is the most expressed. Levels slightly decrease with depth until 2.5 meters, then remain stable with depth. This suggests fermentation could occur throughout the water column, possibly exploiting available dissolved organic carbon, or the reserves accumulated during exposure to light. However, the gene predominantly expressed in the EMP pathway is fructose biphosphate aldolase (FBA). This enzyme converts fructose 1,6 biphosphate into dihydroxyacetone phosphate (DHAP), and is involved in numerous other processes, such as gluconeogenesis, CBB cycle, and responses to various stresses including salt. Indeed, DHAP is the precursor of the glucosylglycerol biosynthesis pathway, which is known to be the major osmoprotective compound in some cyanobacteria (Kirsch et al., 2019). The expression levels of genes involved in glucosylglycerol synthesis are low and constant throughout the water column, in accordance with salinity of the Dziani lake which is stable at all depths. The strategy used by *L. platensis* to cope with hypersalinity is not known, but it has been proved to accumulate glucose at high salt concentration, putatively involved in osmoprotection (Markou et al., 2023). Therefore, gluconeogenesis, whose *eno* and *ppsa* genes both show a strong increase in expression between the surface and 2.5 m depth, could play a role in osmoprotection in the absence of photosynthesis. Overall, FBA expression level could figure complex regulations of and interplay between gluconeogenesis, EMP and CBB pathways. The limited storage capacity of *L. platensis* as well as the aforementioned production of gas vesicles, could be reasons why cell numbers and biomass tend to decrease with depth.

Besides potential uptake of dissolved organic carbon, *P. salinarum* was documented to harbor starch grains that may be used in the absence of light (Aikawa et al., 2012; Cellamare et al., 2018). Metabolism based on non-photosynthetic processes when light is unavailable, including fermentation, was already demonstrated for some other green algae (Catalanotti et al., 2013), and this ability may contribute to the occurrence of *P. salinarum* down to deeper depths, that was also previously documented in the Mono Lake (Phillips et al., 2021). In *P. salinarum*, the EMP and ethanol pathway are the most expressed. The former peaks at 1 m depth, while the latter increases from surface to 1 m. Both are then stable and at similar levels throughout the lower water column. But as for *L. platensis*, FBA is by far the most expressed gene of the EMP pathway, preventing any definitive conclusion about the use of this pathway by *P. salinarum*. Other genes involved in gluconeogenesis are also expressed at 1 m and below, and FBA expression pattern is similar to that of CBB genes. On the other hand, the elevated levels of genes involved in ethanol fermentation are more conclusive and suggest that *P. salinarum* is using ethanol fermentation when located below the oxycline.

## 5 Conclusion

The co-dominance of *Limnospira platensis* and *Picocystis salinarum* observed in lake Dziani Dzaha, in terms of cell numbers, is an unusually documented phenomenon. Investigation of gene expression levels reveals that populations of both taxa are metabolically active down to 16 m depth, even if lakes average depth is much shallower (3.5–4 m). In both species, photosynthesis-related genes are highly expressed in the upper water column, yet with differences that may derive for distinct strategies. Meanwhile *L. platensis* seems to preferentially occupy the upper water column, *P. salinarum* is abundant throughout. Interestingly, the *psb*A gene is by far the most highly expressed by *P. salinarum* throughout the water column, at levels so high that they suggest a possible adaptation to extensive photosynthesis-induced damage on the PS II. Further work should test this hypothesis by addressing the relationship between *psbA* levels, temperature, oxidative stress, light quality and intensity. In the anoxic and aphotic zone, phototrophs are still highly dominant. There, expression of genes involved in fermentation pathways suggests that both phototrophs might be actively metabolizing their stored resources, or locally-available dissolved organic carbon compounds. To which extent do phototrophs circulate between the euphotic and aphotic zones remains to be investigated, yet high expression of at least one gene involved in gas vesicles production in *Limnospira* suggests active anti-sinking strategies and might contribute to the vertical distribution of this species. Overall, the ability to rapidly shift from photosynthesis to fermentation of reserve compounds is certainly key to maintain the co-dominance of both phytoplanktonic species throughout the water column.

## Data availability statement

The original contributions presented in the study are publicly available. This data can be found here: sequences from the metatranscriptomes of the different sampling depths are available under BioProject number PRJNA1037317. Metagenomes from strains are available under Biosample SAMN40550558 (*L. fusiformis*) and SAMN40550586 (*P. salinarum*).

## Author contributions

SD: Conceptualization, Funding acquisition, Investigation, Methodology, Project administration, Resources, Supervision, Validation, Writing – original draft. SH: Conceptualization, Data curation, Formal analysis, Funding acquisition, Investigation, Methodology, Resources, Software, Writing – original draft. J-PB: Conceptualization, Resources, Validation, Writing – original draft. TR: Formal analysis, Investigation, Methodology, Writing – review & editing. MH: Conceptualization, Formal analysis, Funding acquisition, Investigation, Methodology, Project administration, Writing – review & editing. MB: Investigation, Methodology, Resources, Writing – review & editing. PO: Conceptualization, Funding acquisition, Investigation, Methodology, Writing – review & editing. CD: Data curation, Formal analysis, Investigation, Methodology, Writing – review & editing. AW: Data curation, Formal analysis, Writing – review & editing. DJ: Conceptualization, Funding acquisition, Investigation, Methodology, Writing – review & editing. MA: Conceptualization, Data curation, Funding acquisition, Investigation, Methodology, Project administration, Resources, Writing – review & editing. CL: Conceptualization, Formal analysis, Funding acquisition, Investigation, Methodology, Project administration, Resources, Writing – review & editing. HA: Conceptualization, Funding acquisition, Investigation, Methodology, Project administration, Resources, Writing – review & editing. VG: Conceptualization, Funding acquisition, Investigation, Resources, Writing – review & editing. MT: Conceptualization, Data curation, Formal analysis, Funding acquisition, Investigation, Methodology, Project administration, Resources, Writing – review & editing. CB: Conceptualization, Data curation, Formal analysis, Funding acquisition, Investigation, Methodology, Project administration, Writing – original draft.
