# Supplementary material for: Transcriptomic insights into the dominance of two phototrophs throughout the water column of a tropical hypersaline-alkaline crater lake (Dziani Dzaha, Mayotte)
Source: Front Microbiol. 2024 Apr 29;15:1368523. doi: 10.3389/fmicb.2024.1368523 (PMC11089139; doi:10.3389/fmicb.2024.1368523)

Supplementary Figure 1: Calibration curves showing the relationship between cell density and OD_{750} values for *Limnospira platensis* (upper panel) and *Picocystis salinarum* strains (lower panel). Linear regression and R^2 values are indicated for each strain ($p<0.05$).

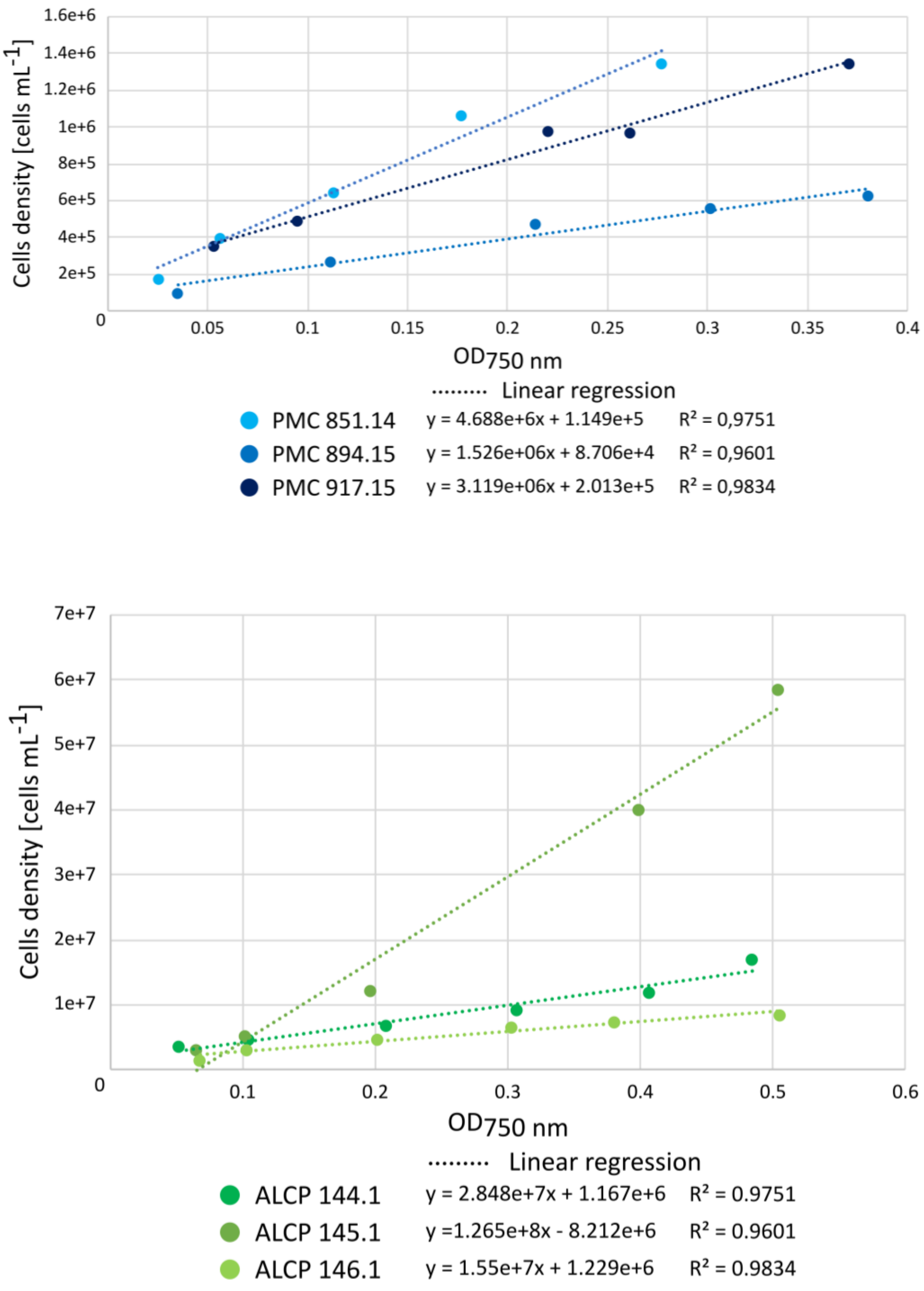

Supplementary Figure 2: Circular map of *Picocystis salinarum* ALCP 144.1 chloroplast genome. The inner circle displays GC content along the genome. The outer circle represents the genes by boxes which size is proportional to the gene length, and colored following gene product function. Genes inside the circle are transcribed counter-clockwise.

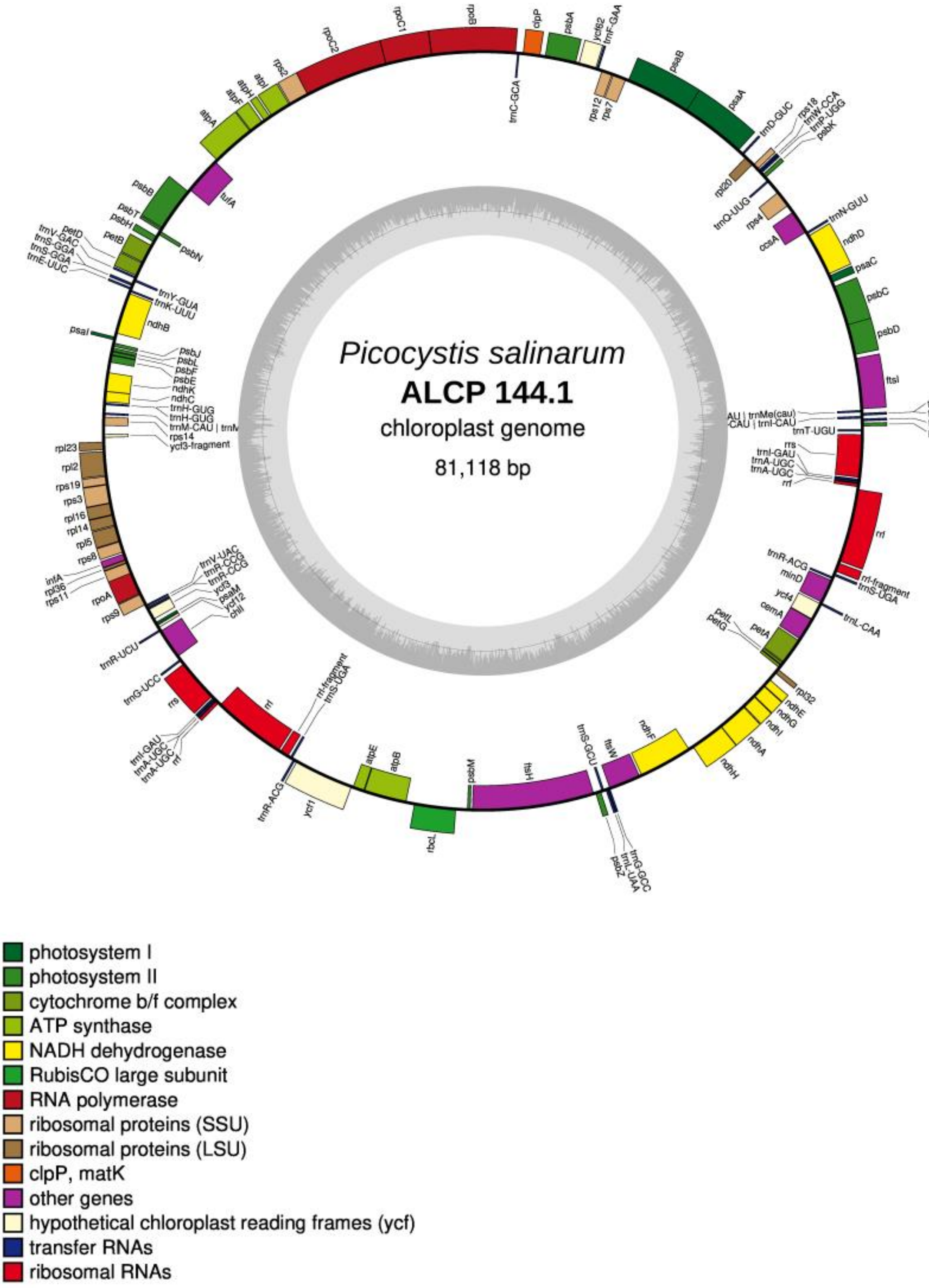

Supplementary Figure 3. Expression levels (in TPM) of *L. platensis* genes involved in gas vesicles formation versus depth.

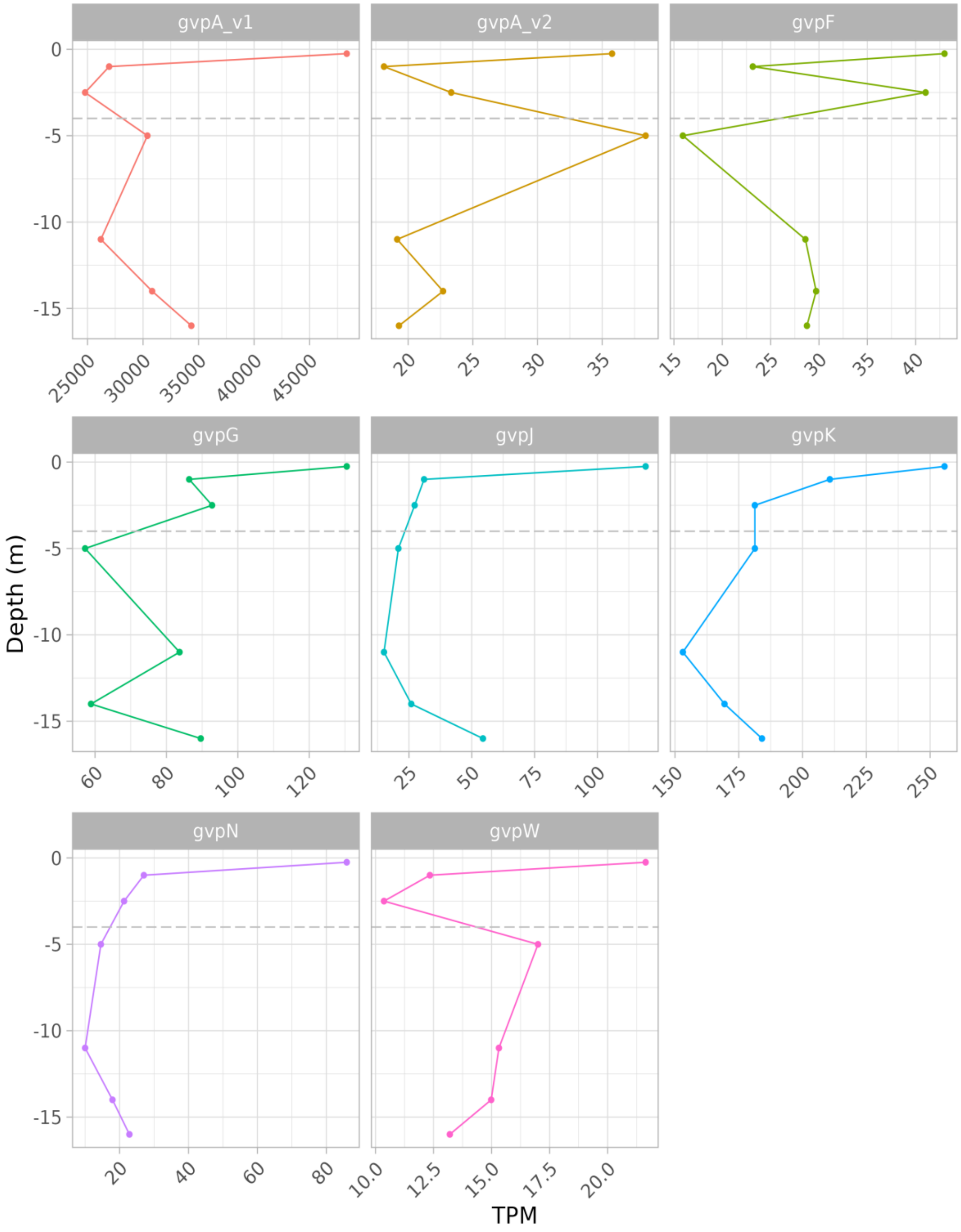

Supplement: Supplementary file 1 [file Presentation_1.pdf]
