# Supplementary material for: Transcriptomic insights into the dominance of two phototrophs throughout the water column of a tropical hypersaline-alkaline crater lake (Dziani Dzaha, Mayotte)
Source: Front Microbiol. 2024 Apr 29;15:1368523. doi: 10.3389/fmicb.2024.1368523 (PMC11089139; doi:10.3389/fmicb.2024.1368523)
Supplement: Supplementary file 3 [file Table_2.docx]

Supplementary Table 2 : Maximum growth rate [µ_max_ d^-1^] and doubling time (T_d_) [d] of *Limnospira platensis* (*L.p*.) strains (PMC 851.14, 894.15 and 917.15) and *Picocystis salinarum* (ALCP 144., 145.1 and 146.1) strains isolated form Dziani Dzaha, depending on light quality.

| Strain | White | | Blue | | Green | | Red | |
| --- | --- | --- | --- | --- | --- | --- | --- | --- |
|  | µ_max_ [d^-1^] | T_d_ (d) | µ_max_ [d^-1^] | T_d_ (d) | µ_max_ [d^-1^] | T_d_ (j) | µ_max_ [d^-1^] | T_d_ (d) |
| *L. p.* PMC 851.14 | 0.095 | 7.3 | 0.019 | 36.6 | 0.110 | 6.4 | 0.091 | 7.6 |
| *L. p.* PMC 894.15 | 0.090 | 7.7 | 0.026 | 26.6 | 0.057 | 12.1 | 0.077 | 9.0 |
| *L. p.* PMC 917.15 | 0.068 | 10.2 | 0.025 | 27.6 | 0.058 | 12.0 | 0.120 | 5.8 |
| *P. s.* ALCP 144.1 | 0.041 | 16.9 | 0.042 | 16.4 | 0.042 | 16.3 | 0.043 | 16.0 |
| *P. s.* ALCP 145.1 | 0.051 | 13.5 | 0.055 | 12.6 | 0.054 | 12.7 | 0.055 | 12.6 |
| *P. s.* ALCP 146.1 | 0.036 | 19.0 | 0.038 | 18.2 | 0.040 | 17.4 | 0.038 | 18.3 |
